# Radiologically measured optic nerve sheath diameter as a predictor of mortality and functional outcomes in stroke patients: A systematic review and meta-analysis

**DOI:** 10.12669/pjms.42.9.16989

**Published:** 2026-09

**Authors:** Wenyan Chen, Jiayuan Zhu

**Affiliations:** 1 Wenyan Chen, Department of Radiology, Huzhou Third Municipal Hospital, the Affiliated Hospital of Huzhou University, Huzhou, Zhejiang Province 313000, P.R. China; 2 Jiayuan Zhu, Department of Radiology, Huzhou Third Municipal Hospital, the Affiliated Hospital of Huzhou University, Huzhou, Zhejiang Province 313000, P.R. China

**Keywords:** Stroke, Mortality, Functional outcome, Ultrasonography, Computed tomography

## Abstract

**Objective:**

Optic nerve sheath diameter (ONSD) has emerged as a non-invasive marker of intracranial pressure and may provide prognostic information in patients with acute stroke. However, studies evaluating its association with mortality and functional outcomes have reported inconsistent findings. This systematic review and meta-analysis aimed to evaluate the prognostic value of radiologically measured ONSD in stroke patients.

**Methodology:**

A systematic search of PubMed, Embase, Scopus, and Web of Science was conducted from database inception to March 10, 2026. Observational studies evaluating ONSD measured by computed tomography or ocular ultrasonography in adult stroke patients were included. Random-effects meta-analysis was performed to pool mean differences (MD) in ONSD between outcome groups and odds ratios (OR) for mortality associated with elevated ONSD.

**Results:**

Sixteen studies were included in the review. Seven studies reported ONSD values for mortality analysis, demonstrating significantly larger ONSD in patients who died compared with survivors (MD 0.50 mm, 95% CI 0.15-0.85; I²=86%). Five studies evaluating functional outcomes showed significantly greater ONSD in patients with poor outcomes (MD 0.60 mm, 95% CI 0.26-0.93; I²≈89%). Three studies reporting risk estimates indicated that elevated ONSD was associated with a higher risk of mortality (OR 5.87, 95% CI 3.11-11.09; I²=64%). Sensitivity analyses and subgroup analyses confirmed the robustness of these findings.

**Conclusions:**

Enlarged ONSD may be associated with increased mortality and poorer functional outcomes in stroke patients. ONSD measurement may serve as a simple, non-invasive adjunct for early prognostic assessment, although further standardised prospective studies are needed.

***Registration No:*** PROSPERO (CRD420261344376).

## INTRODUCTION

Stroke is a major cause of death and long-term disability worldwide. Its management poses significant challenges for both patients and healthcare providers.^1^ Every year, stroke causes loss of life in millions of patients, and several survivors have lasting neurological issues and difficulties with daily functioning.^2^,^3^ Therefore, there is a need to accurately predict mortality and functional outcomes early during stroke in order to allow clinicians make informed clinical decisions, allocate healthcare resources effectively, and also give meaningful prognostic information to patients and their families.

Various clinical and radiological indicators have been examined for their potential to predict stroke outcomes.^4^-^6^ Of these, the National Institutes of Health Stroke Scale (NIHSS) is most commonly used to evaluate the functional outcomes and assess prognosis.^7^ Moreover, neuroimaging based indices, like infarct size, extent of haemorrhage, and early ischemic changes seen in computed tomography (CT) or magnetic resonance imaging (MRI), can also be used to assess prognosis of stroke patients.^8^ Nevertheless, such indices may not be exactly accurate and other factors like intracranial pressure (ICP) elevation and cerebral oedema are usually missed and these are known to impact mortality and functional outcomes.^9^

The optic nerve sheath diameter (ONSD) is recently gaining importance as a promising non-invasive indicator of ICP.^9^ As the optic nerve sheath is continuous with the intracranial subarachnoid space, any variations in the cerebrospinal fluid pressure is transmitted along the nerve. As a result, increases in ICP leads to distension of the optic nerve sheath, which can be noted on ultrasonography, CT, or MRI.^4^ Numerous studies have noted a link between enlarged ONSD and elevated ICP in various neurological conditions, like traumatic brain injury, intracranial haemorrhage, and hypoxic-ischemic encephalopathy.^10^

However, the utility of ONSD for predicting mortality and functional outcomes in such diseases remains uncertain. Some studies indicate that ONSD may help predict mortality and recovery following a stroke, but outcomes have not been consistent.^11^-^13^ To date, no comprehensive systematic review has evaluated the efficacy of ONSD in predicting outcomes after stroke. Given this gap in literature, we undertook the present review to determine whether ONSD can be a reliable marker for mortality and functional outcomes after stroke.

## METHODOLOGY

This systematic review and meta-analysis were conducted in accordance with the PRISMA 2020 guidelines.^14^ Protocol was registered on PROSPERO (CRD420261344376).

### Eligibility criteria:

The eligibility criteria were established in accordance with the PECOS framework. The study population included adult patients (≥18 years) diagnosed with any type of stroke. All patients were to undergo radiological measurement of ONSD, via CT or ocular ultrasound. The exposure group consisted of high ONSD, and the comparator group consisted of low ONSD. The primary outcomes examined were mortality and functional status. Eligible studies included observational studies. The reviewers excluded studies were published as case reports, conference abstracts, or those studies that lacked extractable ONSD data for outcome groups. We also excluded studies with overlapping populations.

### Search strategy:

Two independent reviewers conducted a thorough literature search across PubMed, Embase, Scopus, and Web of Science from the start of each database up to 10 March 2026. There were no language restrictions. Additional articles were searched by manually screening references of relevant studies and via Google Scholar. All search results were imported into EndNote (version 21) for deduplication. Two reviewers then independently conducted a three-step screening process: first removing duplicates, then reviewing titles and abstracts, and finally evaluating full-text eligibility. Any disagreements were resolved through discussion.

### Data extraction:

The following data was collected from the studies: author names, publication years, countries of study, study designs, types of stroke, imaging modalities used for ONSD measurement, sample sizes, age and sex, timing of ONSD measurement, and ONSD values for different outcome groups, including mortality versus survival or poor versus favourable functional outcomes. In addition, reported effect estimates, such as odds ratios (ORs) with 95% confidence intervals (CIs), were extracted if reported. If studies presented ONSD values as medians with interquartile ranges, they were converted to means and standard deviations using methods of Wan et al.^15^ The entire data extraction was conducted independently by two reviewers, and disagreements were resolved through discussion.

### Quality assessment:

The quality and bias risk of the included studies was examined using the Newcastle-Ottawa Scale (NOS).^16^ Scores on the NOS range from 0 to 9, where 7-9 indicate low bias risk, 4-6 indicate moderate risk, and three or below indicate high bias risk. Two reviewers independently conducted the quality assessment, and any disagreements were resolved by consensus.

### Statistical analysis:

This was conducted utilizing R software. For continuous variables, pooled mean differences (MDs) with 95% CIs were calculated to compare ONSD measurements between groups categorised by mortality status and functional outcomes, classified as poor or favourable. When risk estimates were available, pooled ORs with 95% CIs were derived to examine the relationship between elevated ONSD and mortality risk. Adjusted data was used if available, otherwise OR was calculated from raw data. A random-effects meta-analysis, employing the DerSimonian-Laird method, was used to account for potential variability among studies. Heterogeneity across studies was evaluated using Cochran’s Q test and quantified by the I² statistic. To investigate potential sources of heterogeneity, subgroup analyses were performed by geographic location, study design, stroke subtype, and imaging modality used for ONSD measurement. Sensitivity analyses were carried out to check robustness of the findings. Due to the small number of studies, funnel plots were not prepared.

## RESULTS

The study flowchart is shown in Fig.1. Sixteen studies were included in the meta-analysis.^11^-^13^,^17^-^29^ The studies were published between 2017 and 2025 and originated from Asia, Africa, North America, and South America (Table-I). ONSD measurements were obtained using either CT or ocular ultrasound, most commonly at admission or within the first 24 hours of hospitalization. Poor functional outcome was defined as a modified Rankin Scale score of 3-6 after 3-6 months in most studies. Only one study^13^ used the Glasgow Outcome Scale. Risk of bias analysis is presented in Supplementary Table-I. The NOS scores ranged from six to eight.

**Table-I:** Details of included studies.

Study Country	Design	Stroke type	Imaging modality	Sample size	Age (years)	Male (%)	Timing of ONSD	Mortality gro-up ONSD (N)	Survival gro-up ONSD (N)	Poor functional outcome ONSD (N)	Good functional outcome ONSD (N)
Yesilaras 2017 Turkey	CC	Hemorrhagic	CT	61	56	39.3	ED presentation	6.8 ± 0.84 (23)	6.51 ± 1.16 (38)	NR	NR
Seyedhosseini 2019 Iran	PC	Ischemic + hemorrhagic	CT	60	69.67	55	ED presentation	4.4± 0.64 (6)	3.83± 0.56 (54)	NR	NR
Zhao 2019 China	RC	Ischemic + hemorrhagic	Ultrasound	83	NR	NR	At admission	5.5± 0.4 (45)	4.4± 0.5 (38)	NR	NR
Cenik 2021 Turkey	PC	Hemorrhagic	CT	56	58.8	28.6	At diagnosis	5.0 ± 0.74 (22)	4.33 ± 0.74 (34)	NR	NR
Patel 2021 USA	PC	Ischemic + hemorrhagic	Ultrasound	86	67.2	54.7	At admission	6 (26)	5.4 (60)	NR	NR
Xu 2022 China	RC	Hemorrhagic	CT	529	58.1	69.0	Initial CT within 6 h of onset	NR	NR	5.87 ± 0.86 (370)	5.21 ± 0.69 (159)
Zhu 2022 China	RC	Ischemic + hemorrhagic	CT	67	59.7	47.8	24h of admission	6.32± 0.67 (42)	5.65± 0.62 (25)	NR	NR
Batur 2023 Turkey	CC	Ischemic + hemorrhagic	Ultrasound	105	66.5	52.4	At admission	5.42	4.77	NR	NR
Dias 2023 Brazil	PC	Hemorrhagic	Ultrasound	44	62.3	72.7	At hospital admission	5.75± 0.53 (18)	5.42± 0.19 (26)	NR	NR
Hawash 2023 Egypt	RC	Ischemic stroke	Ultrasound	80	NR	48.8	Day 1 after admission	NR	NR	4.47 ± 0.67 (66)	4.04 ± 0.26 (14)
Lee 2024 South Korea	RC	Ischemic stroke	CT	155	NR	57.4	At admission	NR	NR	5.85 ± 0.73 (38)	5.59 ± 0.52 (117)
Vagal 2024 USA	PC	Hemorrhagic	CT	93	64.1	63.4	At admission	NR	NR	6.35± 0.95 (38)	6.17 ± 0.89 (55)
Opare-Addo 2025 Ghana	PC	Ischemic + hemorrhagic	Ultrasound	116	NR	59.6	24h of admission	NR	NR	NR	NR
Silpa 2025 India	PC	Ischemic stroke	Ultrasound	66	55.3	56.1	At admission	NR	NR	5.4 ± 1 (37)	3.9 ± 0.5 (29)
Sivas 2025 Turkey	RC	Ischemic stroke	CT	93	74	52.7	At admission	5.0 ± 0.5 (5)	5.3 ± 0.7 (88)	NR	NR
Zhang 2025 China	PC	Ischemic stroke	Ultrasound	419	66.7	69.7	Within 72h of onset	NR	NR	NR	NR

PC: prospective cohort; RC: retrospective cohort; CT: computed tomography; NR: not reported; ONSD: optic nerve sheath diameter; N: number of patients.ONSD values reported as Mean ± Standard deviation.

**Supplementary Table-I:** Risk of bias analysis.

Study	Selection (4)	Comparability (2)	Outcome (3)	Total score
Seyedhosseini 2019	3	1	2	6
Patel 2021	3	2	2	7
Cenik 2021	3	1	2	6
Xu 2022	4	2	2	8
Zhu 2022	3	2	2	7
Zhao 2019	3	1	2	6
Hawash 2023	3	1	2	6
Batur 2023	3	1	2	6
Dias 2023	3	2	2	7
Lee 2024	3	2	2	7
Vagal 2024	3	2	2	7
Opare-Addo 2025	3	1	2	6
Silpa 2025	3	1	2	6
Sivas 2025	3	2	2	7
Zhang 2025	3	2	2	7
Yesilaras 2017	3	1	2	6

**Fig.1:**
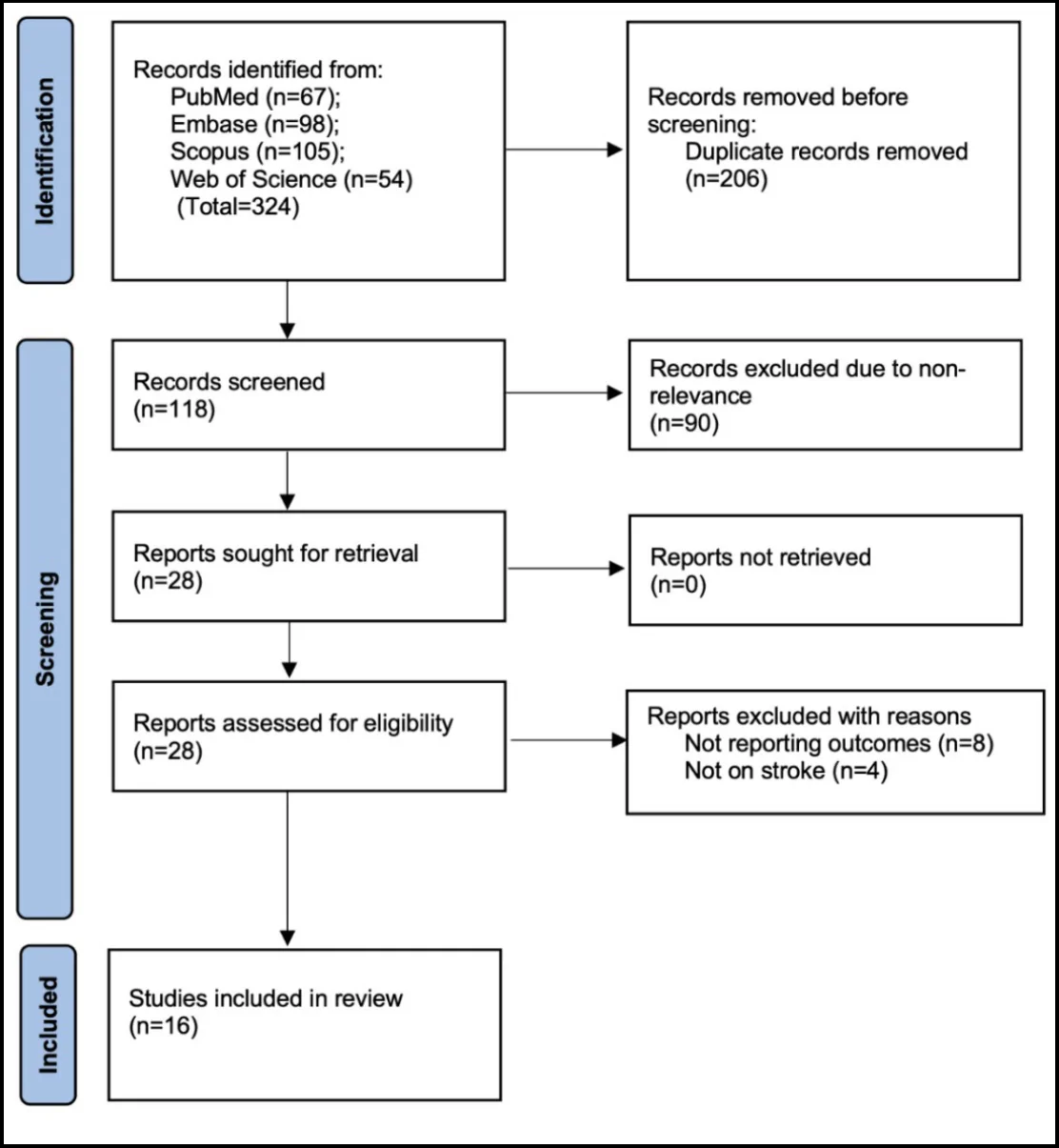
Study flowchart.

Seven studies reported ONSD values for both mortality and survival groups and were included in the pooled analysis. Meta-analysis demonstrated that patients who died had significantly larger ONSD compared with survivors, with a pooled MD of 0.50 mm (95% CI 0.15-0.85). Substantial heterogeneity was observed across the included studies (I² = 86%). (Fig.2).

**Fig.2:**
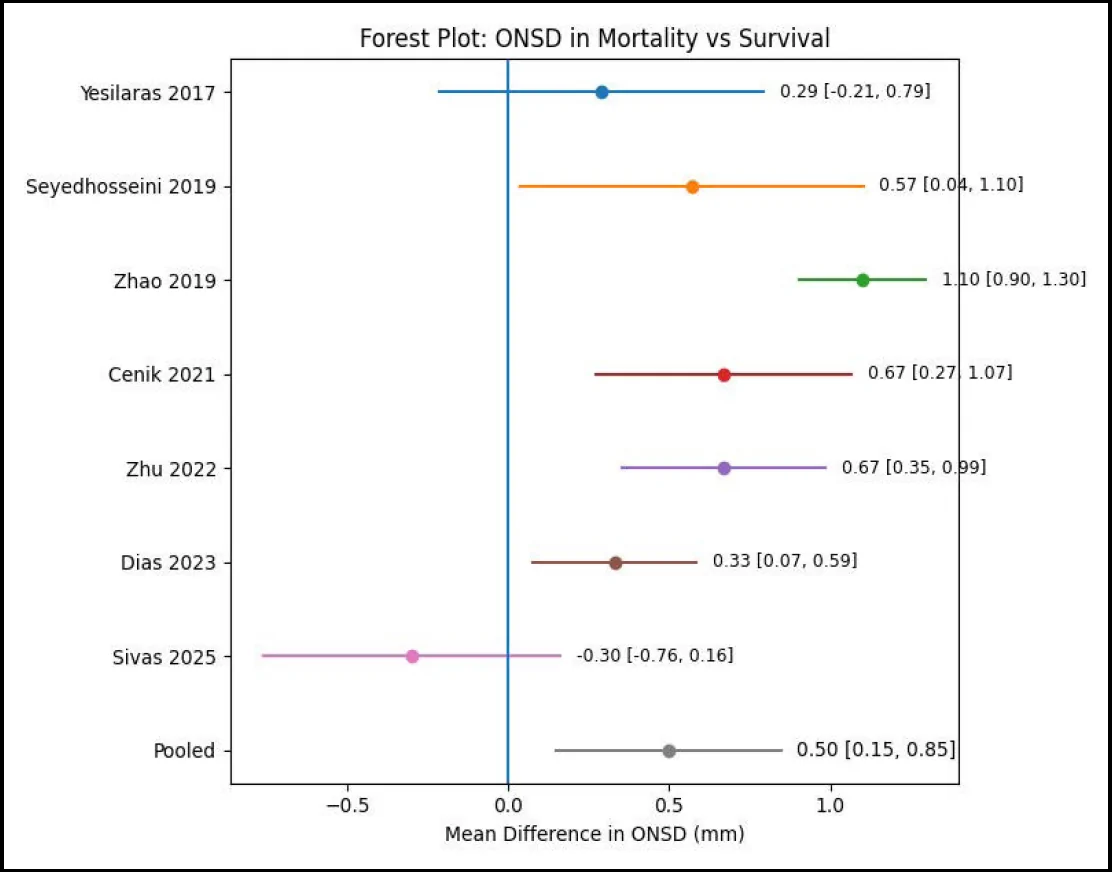
Meta-analysis of the difference in ONSD between mortality and survivor groups.

Five studies provided data comparing ONSD between patients with poor functional outcomes and those with favourable outcomes. Meta-analysis showed that ONSD was significantly larger in patients with poor functional outcomes compared with those with good outcomes, with a pooled mean difference of 0.60 mm (95% CI 0.26-0.93). Heterogeneity across studies was considerable (I² ≈ 89%) (Fig.3).

**Fig.3:**
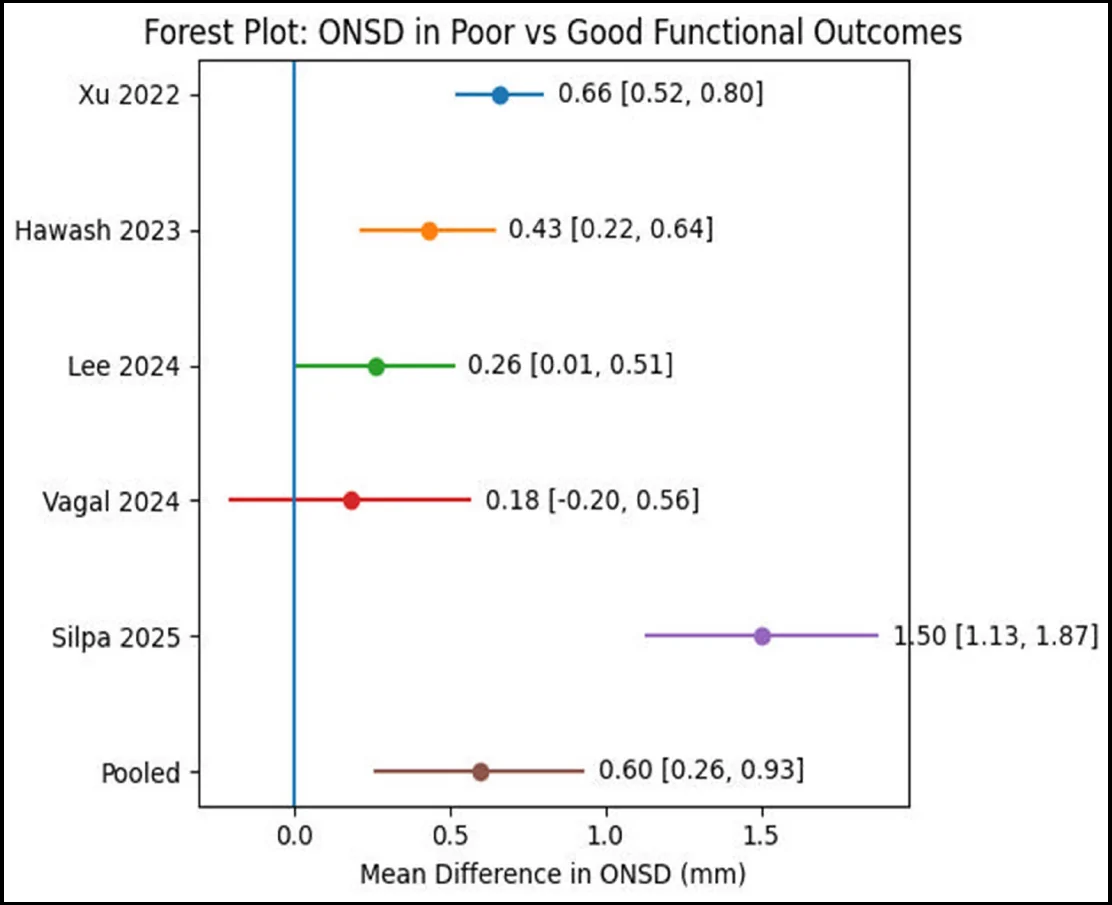
Meta-analysis of the difference in ONSD between good and poor functional outcomes.

Three studies reported odds ratios evaluating the association between elevated ONSD and mortality. Meta-analysis demonstrated that increased ONSD was significantly associated with a higher risk of mortality, with a pooled odds ratio of 5.87 (95% CI 3.11-11.09). Heterogeneity was moderate (I² = 64%) (Fig.4).

**Fig.4:**
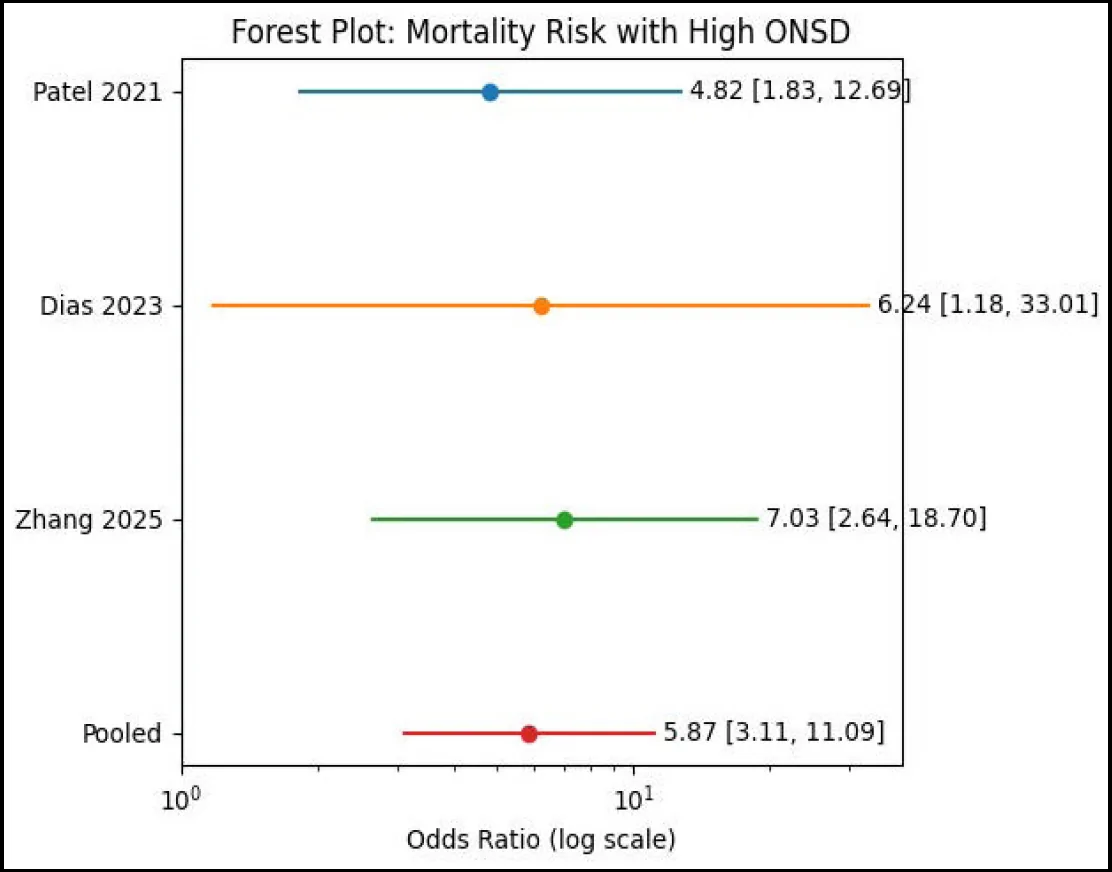
Meta-analysis of the association between ONSD and mortality.

Subgroup analyses results are presented in Table-II and Table-III. Most results remained stable on subgroup analysis. However, there were some non-significant results, especially in subgroups with a small number of studies.

**Table-II:** Subgroup meta-analysis of optic nerve sheath diameter between mortality and survival groups.

Subgroup variable	Subgroup	No. of studies	Mean difference (mm)	95% CI	I^2^ (%)
Continent	Asia	6	0.53	0.12, 0.93	86
	South America	1	0.33	0.07, 0.59	NA
Study design	Case-control	1	0.29	−0.21, 0.79	NA
	Prospective cohort	3	0.46	0.24, 0.68	10
	Retrospective cohort	3	0.52	−0.19, 1.23	94
Stroke type	Hemorrhagic	3	0.41	0.20, 0.63	11
	Mixed stroke	3	0.83	0.47, 1.18	72
	Ischemic	1	-0.3	−0.76, 0.16	NA
Imaging modality	CT	5	0.39	0.04, 0.75	71
	Ultrasound	2	0.72	−0.04, 1.47	95

NA: not applicable; CI: confidence intervals; CT: computed tomography.

**Table-III:** Subgroup meta-analysis of optic nerve sheath diameter based on functional outcomes.

Subgroup variable	Subgroup	Studies	Mean difference (mm)	95% CI	I^2^ (%)
Continent	Africa	1	0.43	0.22, 0.64	-
	Asia	3	0.79	0.25, 1.32	93.24
	North America	1	0.18	-0.20, 0.56	-
Design	Prospective	2	0.84	-0.45, 2.13	95.76
	Retrospective	3	0.47	0.23, 0.70	77.02
Stroke type	Hemorrhagic	2	0.45	-0.01, 0.92	81.27
	Ischemic	3	0.71	0.09, 1.33	93.69
Modality	CT	3	0.40	0.06, 0.73	81.91
	Ultrasound	2	0.95	-0.09, 2.00	95.87

CI: confidence intervals; CT: computed tomography.

Sensitivity analyses for the mortality analysis showed that the exclusion of individual studies did not alter the significance of the effect size, with the MD ranging from 0.39 to 0.63 mm. Similarly, for the functional outcome analysis, the pooled MD ranged from 0.42 to 0.69 mm after sequentially excluding individual studies, with no change in the significance of results.

## DISCUSSION

This review and meta-analysis found that elevated ONSD may predict higher mortality and poorer functional outcomes in stroke patients. Deceased patients had larger ONSD, with a MD of 0.50 mm, and those with poor recovery also had significantly larger ONSD, with a MD of 0.60 mm. Elevated ONSD was linked to a higher risk of mortality, with an OR of 5.87. However, the pooled MD for mortality functional outcome were modest and approach the order of inter-method/inter-observer variability previously reported for ONSD. Moreover, no standardised ONSD threshold was applied consistently across studies, with cut-offs varying by modality, population, and outcome definition. These group-level differences should therefore be regarded as evidence of association rather than a validated diagnostic threshold, and studies reporting sensitivity, specificity, and ROC-derived cut-offs are needed.

The prognostic significance of ONSD found in our review is consistent with prior research on other acute neurological and critical care patients. Zhang et al. and Hong et al.^30^ in their meta-analysis of six studies with 516 patients suffering from traumatic brain injury (TBI), showed that increased ONSD was strongly linked to a higher risk of mortality. The study reported a pooled OR of 21.86, an area under the curve (AUC) of 0.87, a sensitivity of 86%, and a specificity of 78%. Another meta-analysis by Zhang et al.^31^ which included eight studies and 473 patients with post-cardiac arrest found that ONSD was significantly associated with poor neurological outcomes (OR 15.62), with an AUC of 0.87 and a very high specificity of 0.94.

Altınsoy et al. and Bayhan et al.^32^ in their study have also shown a statistically significant positive association between MRI-measured ONSD and mortality rates in a group of 102 intensive care unit patients who had cerebrovascular events (p = 0.002). The prognostic relevance of ONSD is also seen in diseases other than neurological conditions. For example, Kus et al.^33^ in their study on 217 patients diagnosed with acute decompensation of cirrhosis found that CT-measured ONSD was independently associated with mortality and disease severity. Moreover, ONSD was shown to have the highest predictive accuracy among all evaluated variables. The results of these studies, along with the present review, indicate that ONSD has prognostic value in different types of acute brain injury. However, the magnitude of the effect appears to vary, possibly due to the variations in underlying pathophysiology of ICP.

A major limitation of this review is that notable statistical heterogeneity was noted in the analysis. This variability among studies is expected given their methodological and clinical differences. There may have been several sources of heterogeneity that include variations in stroke subtype (ischemic, haemorrhagic), imaging methods (CT versus ocular ultrasound), timing of ONSD measurement based on the symptom onset, measurement thresholds and techniques used, outcome definitions, and patient characteristics like stroke severity and comorbidities. Although sensitivity analyses demonstrated robustness of the main findings, the high heterogeneity noted in the analysis warrants caution in interpreting the results. Also, the pooled estimates may not represent a single underlying effect size.

A comprehensive subgroup analysis was performed to investigate the sources of heterogeneity. In subgroup analyses of mortality, it was noted that the pooled MD remained statistically significant in Asian continent subgroup, studies having prospective cohort design, patients with haemorrhagic or mixed stroke subtypes, and in studies utilising CT imaging. However, the results were non-significant in subgroups including only a single study. High heterogeneity was also noted in retrospective cohort studies and ultrasound-modality subgroups. In the subgroup analysis for functional outcomes, the results showed significant associations in those studies from African and Asian geographic regions, retrospective study designs, and including both ischemic and haemorrhagic stroke subtypes, and those using CT imaging. Importantly, studies conducted in North America and those using ultrasound assessments failed to show statistical significance. It is crucial to interpret the non-significant results in some subgroups carefully, as they might be due to the small number of studies in each group, rather than a non-significant effect. Further studies which are adequately powered for each subgroup are needed to confirm these findings.

Ischemic and hemorrhagic stroke differ in pathophysiology, which can affect both the magnitude and timing of ONSD elevation. Haemorrhagic stroke leads to rapid mass effect from the haematoma, whereas ischemic oedema evolves over 24–72 hours. This may explain the divergent subgroup findings: in the mortality analysis, hemorrhagic stroke studies showed a significant difference while the single ischemic study did not, whereas in the functional outcome analysis the ischemic subgroup was significant and the hemorrhagic subgroup was not. Considering these limitations, the pooled overall estimates should not be assumed as equal across stroke subtypes, and these findings, are best regarded as hypothesis-generating rather than conclusive.

Both CT and ocular ultrasound have been used to measure ONSD, and prior research has failed to find any significant difference between the two methods. A prospective study with 199 emergency department patients has found high agreement between ultrasound- and CT-based ONSD measurements, with a high intraclass correlation coefficient of 0.785.^34^ A meta-analysis of patients with intracranial hypertension has shown that ultrasound and CT measurements result in similar accuracy, suggesting they could be used interchangeably for diagnosing increased ICP.^35^ Indeed, each imaging modality has its unique advantages and limitations. CT has broad availability, is non-invasive, and offers quick assessment. But it also requires patient transport, exposes patients to radiation, and isn’t indicated for repeated measurements.

On the other hand, ultrasound is a quick, accessible bedside technique that avoids radiation and can detect ICP changes rapidly. However, it is operator-dependent and may be unsuited after ocular trauma, and varies with threshold settings.^34^,^35^ In stroke cases, both methods are effective at or near admission, and since CT is routinely part of stroke diagnostics, CT-based ONSD measurement should not add an extra burden and can be easily added into standard protocols. Ultrasound is especially valuable in settings where frequent or bedside monitoring is needed, especially in resource-limited settings without CT access, or for patients who cannot to be transported safely.^17^

### Limitations:

First, the number of studies contributing to each pooled analysis was relatively small, limiting the statistical power of subgroup analyses and this may introduce imprecision into the study results. Second, the substantial heterogeneity was seen which persisted on subgroup analysis, indicating that other known and unknown confounders may have affected the results. Third, residual confounding from unmeasured clinical variables is possible in the included study designs. It is important to note that, as included studies were largely unadjusted for variables such as stroke severity, infarct volume, hematoma size, and NIHSS score, these findings reflect an association rather than confirming ONSD as an independent prognostic predictor. Fourth, the majority of included studies originated from Asia. This limits generalisability of evidence to other geographic and ethnic populations. Fifth, ONSD measurement thresholds and methodologies varied across studies, this limited a definitive recommendation for a single diagnostic threshold. Lastly, a pooled analysis of OR was only possible for mortality, with only three studies. Similar analysis could not be conducted for functional outcomes due to scarce data.

The results of this meta-analysis have implications in clinical practice. Our results indicate that incorporating ONSD into early stroke risk assessment protocols is feasible and potentially important, especially in emergency and critical care setting, where rapid and accurate prognostic information can guide decisions about monitoring intensity, care escalation, and discussions with families about the goals of treatment. Nevertheless, one limitation of ONSD is that, measurements may vary with the technique and operators and even small errors may lead to erroneous results. Also, the present evidence does not provide the exact threshold for ONSD and more evidence is needed on this aspect before it can be used in clinical practice.

However, several important questions persist. Future prospective studies with adequate power are needed to determine modality-specific and stroke subtype-specific ONSD thresholds. Such studies should provide clear sensitivity and specificity data, ideally following standardised measurement protocols. Studies should also use multivariate analyses that include ONSD with other important markers like NIHSS scores, infarct volume, and hematoma characteristics, in order to assess if ONSD provides added independent prognostic information beyond the current clinical and radiological markers. Serial data of ONSD over the first 24 to 72 hours after admission is needed to assess the evolving nature of cerebral oedema and its connection with mortality and functional outcomes. Finally, studies from various geographic, ethnic, and healthcare settings are needed to verify the wider applicability of the current findings.

## CONCLUSIONS

Evidence suggests that ONSD could be a significant predictor of both mortality and functional outcome in stroke patients. An enlarged ONSD at or near admission is associated with a substantially higher risk of death and poor neurological recovery, which supports its use as a simple, non-invasive prognostic marker in the clinical assessment of acute stroke. However, as all included studies were observational and largely unadjusted for variables such as stroke severity, infarct volume, or NIHSS score, this evidence reflects an association rather than an independent predictive role. High heterogeneity of included studies and limited data are important limitations that prevent firm conclusions.
